# Chromatin Remodeling Protein ZmCHB101 Regulates Nitrate-Responsive Gene Expression in Maize

**DOI:** 10.3389/fpls.2020.00052

**Published:** 2020-02-13

**Authors:** Xinchao Meng, Xiaoming Yu, Yifan Wu, Dae Heon Kim, Nan Nan, Weixuan Cong, Shucai Wang, Bao Liu, Zheng-Yi Xu

**Affiliations:** ^1^ Key Laboratory of Molecular Epigenetics of the Ministry of Education (MOE), Northeast Normal University, Changchun, China; ^2^ School of Agronomy, Jilin Agricultural Science and Technology University, Jilin, China; ^3^ Department of Biology, Sunchon National University, Sunchon, South Korea; ^4^ College of Life Sciences, Linyi University, Linyi, China

**Keywords:** chromatin remodeler, nitrate response, transcriptional regulation, nitrate transporter, maize

## Abstract

Nitrate is the main source of nitrogen for plants and an essential component of fertilizers. Rapid transcriptional activation of genes encoding the high-affinity nitrate transport system (HATS) is an important strategy that plants use to cope with nitrogen deficiency. However, the specific transcriptional machineries involved in this process and the detailed transcriptional regulatory mechanism of the core HATS remain poorly understood. ZmCHB101 is the core subunit of the SWI/SNF-type ATP-dependent chromatin remodeling complex in maize. RNA-interference transgenic plants (*ZmCHB101-RNAi*) display abaxially curling leaves and impaired tassel and cob development. Here, we demonstrate that ZmCHB101 plays a pivotal regulatory role in nitrate-responsive gene expression. *ZmCHB101-RNAi* lines showed accelerated root growth and increased biomass under low nitrate conditions. An RNA sequencing analysis revealed that ZmCHB101 regulates the expression of genes involved in nitrate transport, including *ZmNRT2.1* and *ZmNRT2.2*. The NIN-like protein (NLP) of maize, ZmNLP3.1, recognized the consensus nitrate-responsive *cis*-elements (NREs) in the promoter regions of *ZmNRT2.1* and *ZmNRT2.2*, and activated the transcription of these genes in response to nitrate. Intriguingly, well-positioned nucleosomes were detected at NREs in the *ZmNRT2.1* and *ZmNRT2.2* gene promoters, and nucleosome densities were lower in *ZmCHB101-RNAi* lines than in wild-type plants, both in the absence and presence of nitrate. The ZmCHB101 protein bound to NREs and was involved in the maintenance of nucleosome occupancies at these sites, which may impact the binding of ZmNLP3.1 to NREs in the absence of nitrate. However, in the presence of nitrate, the binding affinity of ZmCHB101 for NREs decreased dramatically, leading to reduced nucleosome density at NREs and consequently increased ZmNLP3.1 binding. Our results provide novel insights into the role of chromatin remodeling proteins in the regulation of nitrate-responsive gene expression in plants.

## Introduction

Maize (*Zea mays*) is one of the most important crops in the world. Approximately 70% of the kernel weight in maize is composed of starch, which is the main source of energy in the human and animal diet. To maximize the yield of maize crop in the field, large quantities of nitrogenous fertilizers are added to the soil during cultivation. Over the past several decades, application of nitrogen (N) fertilizer has significantly increased maize production (Zhang et al., 2011; Sun and Zheng, 2015; Alvarez et al., 2019). As one of the most important macronutrients for plants, N is required for the biosynthesis of proteins, nucleic acids, chlorophyll, ATP, alkaloids, and hormones (Tills and Alloway, 1981; Shadchina and Dmitrieva, 1995; Lam et al., 1996). Therefore, N deficiency limits plant growth and development, thereby reducing crop yield (Chen et al., 2016). However, crops utilize only approximately 30% of the applied N fertilizer (Raun and Johnson, 1999; Sultan, 2003), while the remaining N causes environmental pollution *via* gaseous emission, fertilizer leaching, surface runoff, and denitrification (Good et al., 2004).

In the soil, N is present in two main forms, nitrate and ammonia, both of which are crucial for plant growth and root development (Stitt and Feil, 1999; Zhang et al., 1999). The local stimulatory effect of nitrate on lateral root elongation results from its function as a signal rather than a nutrient (Zhang et al., 1999). Plant nitrate uptake is mediated by low- and high-affinity transport systems that function at high and low external nitrate concentrations, respectively (Huang et al., 1999). In the model plant *Arabidopsis thaliana*, AtNPF6.3 acts as a unique nitrate transporter that mediates both low- and high-affinity nitrate uptake (Ho et al., 2009; Parker and Newstead, 2014). The AtNRT2.1 protein plays a major role in high-affinity nitrate uptake, whereas AtNRT2.2 makes a relatively small contribution (Li et al., 2007). In addition, the nitrate transporter, AtNRT2.5, facilitates nitrate uptake and remobilization in N-starved *A. thaliana* (Lezhneva et al., 2014). Under nitrate-deficient conditions, the activities of high-affinity nitrate transporters and the transcript levels of *AtNRT2.1* and *AtNRT2.2* increase rapidly with nitrate supply (Zhuo et al., 1999; Okamoto et al., 2003); however, both of these genes are subsequently repressed upon prolonged exposure to sufficient nitrate. Restoring nitrate supply stimulates the nitrate uptake capacity of plants; however, accumulation of nitrate and its assimilatory products, including amino acids, in plant cells reduces the expression of *NRT2* genes, and consequently the nitrate uptake capacity of plants (Zhuo et al., 1999; Vidmar et al., 2000). These data suggest the existence of an underlying mechanism that regulates nitrate uptake in accordance with the N demand (Forde, 2002). In maize, an increase in *ZmNRT2.1* and *ZmNRT2.2* transcript levels activates the nitrate uptake capacity (Sabermanesh et al., 2017); however, the mechanism of *ZmNRT* gene transcription regulation remains unclear.

Chromatin remodeling complexes (CRCs) play pivotal roles in nucleosome sliding and occupancy by controlling ATP-dependent alterations in histone-DNA contacts (Peterson and Workman, 2000; Gangaraju and Bartholomew, 2007; Clapier and Cairns, 2009; Narlikar, 2010). The SWITCH (SWI)/SUCROSE NONFERMENTING (SNF) complexes are multi-subunit complexes that contain more than eight proteins (Sarnowska et al., 2016). Based on the type of SNF2 family ATPase subunits, the ATP-dependent CRCs are divided into four subfamilies: SWI2/SNF2, IMITATION SWITCH (ISWI), Mi-2/Chromodomain-Helicase-DNA (CHD)-binding protein (Mi-2/CHD), and INO80 (Sarnowska et al., 2016). Previous studies revealed that SWI3 proteins, the core components of the SWI/SNF CRCs, play essential roles in plant growth and development (Sarnowski et al., 2005; Yu et al., 2016). The *AtSWI3* genes regulate root elongation and leaf and reproductive organ development (Sarnowski et al., 2005). Mutations in either *AtSWI3A* or *AtSWI3B* cause developmental arrest of the embryo at the globular stage, and mutation of *AtSWI3B* leads to the death of macrospores and microspores (Sarnowski et al., 2005; Hurtado et al., 2006). Furthermore, mutations in *AtSWI3D* lead to severe dwarfism and alterations in the number and development of flower organs (Zhou et al., 2003; Sarnowski et al., 2005). The maize SWI3 protein, ZmCHB101, plays an essential role in leaf development and dehydration and abscisic acid responses (Yu et al., 2016; Yu et al., 2018; Yu et al., 2019); however, it is unknown whether SWI/SNF complexes participate in nitrate responses.

In this study, we found that knockdown of *ZmCHB101* expression in maize accelerated root growth and increased biomass under low nitrate conditions. In addition, we found that ZmCHB101 regulates the expression of genes involved in nitrate transport, including *ZmNRT2.1* and *ZmNRT2.2*. Our results also demonstrate that the NIN-like protein (NLP) in maize, ZmNLP3.1, recognizes nitrate-responsive *cis*-elements (NREs) in the promoters of the *ZmNRT2.1* and *ZmNRT2.2* genes, and it activates the expression of these genes in response to nitrate. Intriguingly, well-positioned nucleosomes were detected at NREs, and nucleosome densities were lower in *ZmCHB101-RNAi* transgenic maize lines than in wild-type (WT) plants, both in the absence and presence of nitrate. In the absence of nitrate, ZmCHB101 bound to the NREs and maintained the nucleosome occupancies at these sites, which may impact the binding of ZmNLP3.1. However, in the presence of nitrate, the binding affinity of ZmCHB101 for NREs decreased dramatically, thus reducing the nucleosome density at NREs and consequently increasing the binding of ZmNLP3.1 to these sites.

## Materials and Methods

### Plant Material and Growth Conditions

*ZmCHB101-RNAi* lines, RS1 and R101, have been described previously (Yu et al., 2016), in which *ZmCHB101* transcript levels were approximately 7% and 16% of that in the WT, respectively. Seeds of the WT and *ZmCHB101-RNAi* lines were sterilized using 1% sodium hypochlorite and incubated on moist filter paper at 28°C for 3 days for germination. Uniform seedlings were chosen and transferred to hydroponic culture in an environmentally controlled chamber with continuous ventilation for 4 days to deplete the nutrients in seeds. Subsequently, seedlings were removed from endosperms and incubated in modified Hoagland's nutrient solution (Li et al., 2015) containing 0 mM nitrate for 1 day under constant aeration. To determine the effect of nitrate induction, seedlings were grown in Hoagland's nutrient solution containing 0, 0.5, 1, 5, or 15 mM nitrate at 23°C day/18°C night temperature under 16 h light/8 h dark conditions for 5 days. The nutrient solution was renewed daily. Morphological parameters of lateral roots were analyzed using the WinRHIZO software (Regent Instruments Canada Inc., Canada). The experiments were repeated three times, and each experiment was performed using 20 plants per genotype. To perform long-term low nitrate induction, germinated seeds were planted in sand and watered with Hoagland's nutrient solution containing 0.5 or 15 mM nitrate for 6 weeks. To conduct RNA sequencing (RNA-Seq) analysis, total RNA was extracted from the roots of seedlings cultured in nitrate-free nutrient solution for 7 days and then treated with Hoagland's nutrient solution containing 0.5 mM nitrate for 0 or 2 h. Three independent replicates were performed for each sample. The same conditions were used for preparing samples for chromatin immunoprecipitation (ChIP) assay, followed by quantitative PCR (qPCR).

### Metabolite Analyses and Enzymatic Assays

*ZmCHB101-RNAi* lines and WT grown in Hoagland's nutrient solution containing 0.5 or 15 mM nitrate were used for metabolite and enzymatic assays. The amount of total N was measured using Elementar Isoprime 100 vario EL cube (Elementar, German). The amount of nitrate was estimated using Smartchem450 automatic chemical analyzer (Unityscientific, USA). The chlorophyll content of plants was measured as described previously (Yang et al., 2014). Soluble protein content was determined using the Plant Soluble Protein ELISA Kit (Jonln, China). The activity of nitrate reductase (NR), nitrite reductase (NIR), and glutamine synthetase (GS) was analyzed using the NR, NIR, and GS ELISA kits (Plant), respectively (Jonln, China).

### Bioinformatics Analyses of RNA-Seq Data

Total RNA was isolated from seedling roots using TRIzol Reagent (Invitrogen, USA), according tothe manufacturer's protocol. Three biological replicates of each sample were used for RNA-Seqlibrary construction and sequenced on the HiSeq2000 platform (Illumina, USA). The raw data werecleaned by removing adaptor sequences and low-quality reads using FASTX-Toolkit version 0.0.13(http://hannonlab.cshl.edu/fastx_toolkit/). At least 110 million clean reads were obtained per library (**Supplementary Table S1**). The clean reads were mapped onto the maizereference genome, B73 RefGen_v3, using Hisat2 (http://ccb.jhu.edu/software/hisat2/index.shtml) with default parameters. The number of Fragments Per Kilobase of transcript per Million mapped reads(FPKM) was used to determine the transcription level of each gene using Cuffdiffv2.0.1. Genes with|log_2_fold-change (FC)| > 1 and false discovery rate (FDR) < 0.05 were identified asdifferentially expressed genes (DEGs). Gene Ontology (GO) analysis of all DEGs was performed using the web-based agriGO tool (http://systemsbiology.cau.edu.cn/agriGOv2/). Singular enrichment analysis (SEA) was used for GO enrichment analysis on agriGO. The R package was used to manage, integrate, and visualize the RNA-Seq data.

### Plasmid Construction

The coding sequence (CDS) of *ZmNLP3.1* was amplified from a cDNA library by PCR using ZmNLP3.1-F/R gene-specific primers. The CDS of *ZmCHB101* was amplified, as described previously (Yu et al., 2018). To generate a fusion construct of *ZmNLP3.1* with *glutathione S-transferase* (*GST-ZmNLP3.1*), the full-length CDS of *ZmNLP3.1* was cloned into the *pGEX-4T-1* vector using *Sma*I and *Not*I restriction sites. To generate the *ZmNLP3.1* overexpression construct, the *ZmNLP3.1* CDS was cloned downstream of the Cauliflower mosaic virus *35S* promoter in the *pCsV1300* vector using *Xba*I and *Cla*I sites, thus generating the *pro35S:ZmNLP3.1* construct. To generate dual FLAG epitope tagged *ZmNLP3.1* and *ZmCHB101* overexpression constructs (*pro35S:ZmNLP3.1-2×FLAG* and *pro35S:ZmCHB101-2×FLAG*), the CDSs of *ZmNLP3.1* and *ZmCHB101* were cloned into the *pCsV1300* vector separately using *Xba*I and *Cla*I sites. To generate luciferase reporter (LUC) constructs of *ZmNRT2.1* and *ZmNRT2.2* (*proZmNRT2.1:LUC* and *proZmNRT2.2:LUC*), a mutant copy of *ZmNRT2.1* or *ZmNRT2.2* promoter (1 kb) carrying AAAAAACCN_10_CCAAA or GAAAAAAGN_10_GAAAG substitution, respectively, was amplified using the *ZmNRT2.1-MPro-*F/R or *ZmNRT2.2-MPro-*F/R primer pair and inserted upstream of the *LUC* reporter gene; constructs containing an intact copy of each promoter upstream of the *LUC* gene were also generated using the *ZmNRT2.1-Pro-*F/R or *ZmNRT2.2-Pro-*F/R primer pair. To generate *proZmUBQ2*:*GUS* construct, *ZmUBQ2* (*GRMZM2G419891*) promoter sequence was amplified using a sequence-specific primer pair (*ZmUBQ-Pro-*F/R) and cloned in the *pCAMBIA3301* vector upstream of the *β-glucuronidase* (*GUS*) gene using *Nco*I and *Pst*I sites. The sequences of these primers are listed in **Supplementary Table S2**.

### Quantitative Real-Time PCR (qRT-PCR)

Total RNA (2 µg) was used to synthesize cDNA with TransScript One-Step gDNA Removal and cDNA Synthesis SuperMix (Transgen Biotech). The qRT-PCR assay was performed using THUNDERBIRD SYBR qPCR Mix (TOYOBO) on the ABI real-time PCR detection system, according to the manufacturer's instructions (ABI StepOnePlus, USA). Three biological replicates in qRT-PCR analysis were performed and each biological replicate was conducted using three technical replicates. The maize *Actin 1* (*ZmACT1*) gene was used as an internal reference. Primers used for qRT-PCR are listed in **Supplementary Table S2**.

### Transient Expression in Protoplasts

Plasmid DNA (20 μg) was used to transfect 200 μl of maize protoplasts (2 × 10^5^ cells ml^-1^), as described previously (Yoo et al., 2007; Yu et al., 2018). To obtain nitrate-free protoplasts, maize seedlings were watered with nitrate-free Hoagland's nutrient solution (pH 6) containing 0.1% MES, 1% sucrose, 2.5 mM ammonium succinate, and 0.5 mM glutamine and incubated in the dark at 23°C for 15–20 days. To examine the expression levels of nitrate-responsive genes, the isolated maize protoplasts were incubated in W5 solution (0.2 mM MES, 154 mM NaCl, 125 mM CaCl_2_, and 5 mM KCl) for 12 h and then transferred into W5 solution supplemented with 0.5 mM nitrate for 2 h. The protoplasts were collected by centrifugation at 100 × *g* for 1 min and then used for qRT-PCR or ChIP assay, as described previously (Yu et al., 2018).

### ChIP Assay

For H3, H3K4me3 and H3K27me3 ChIP-qPCR assays, root tissues of maize seedlings treated with 0.5 mM nitrate for 0 or 2 h were collected and crosslinked in 1% formaldeyde. ChIP-qPCR was performed as described previously (Huang et al., 2012; Yu et al., 2018). Briefly, chromatin was isolated and sheared to 200–800 bp with the M220 Focused-ultrasonicator (Covaris). And soluble protein was incubated with H3 (Abcam, ab1791), H3K4me3 (Abcam, ab8580), or H3K27me3 (Millipore, 17622) antibody at 4°C. To perform ZmNLP3.1 and ZmCHB101 ChIP-qPCR assays, protoplasts isolated from 15-day-old nitrate-free seedlings were used, as described previously (Huang et al., 2012), with some modifications. The isolated maize protoplasts were transfected with the pro35S:ZmNLP3.1-2×FLAG or pro35S:ZmCHB101-2×FLAG construct, incubated in W5 solution for 12 h, and then treated with or without 0.5 mM nitrate for 2 h. The protoplasts were collected and subjected to crosslinking in 1% formaldehyde. The isolated chromatin was sheared to 200–800 bp fragments using an M220 Focused-ultrasonicator (Covaris, USA). The soluble chromatin was incubated with anti-FLAG antibody (MBL, D153-8) or serum overnight at 4°C. The immunoprecipitates were reverse crosslinked by heating the sample at 65°C for 8 h, and DNA was extracted using the phenol-chloroform extraction method. The ZmNRT2.1 and ZmNRT2.2 gene promoter fragments were amplified by qPCR using sequence-specific primers (**Supplementary Table S2**). The ZmACT1 gene was used as a negative control.

### Electrophoretic Mobility Shift Assay (EMSA)

The fusion construct *GST-ZmNLP3.1* or the plasmid expressing GST alone was transformed into Escherichia coli BL21 (DE3) cells. The GST-ZmNLP3.1 and GST proteins were purified with glutathione beads (Xu et al., 2013), according to the manufacturer's protocol. Briefly, 5'-biotinylated probes were synthesized and labeled with biotin by Sangon Biotechnology. Double-stranded probe (50 fmol) was mixed with each purified protein separately in binding buffer and incubated for 10 min. The reaction mixtures were subjected to electrophoresis on a native 6% polyacrylamide gel in 0.5× TBE buffer. DNA in the gel was transferred to a positive charged nylon membrane and detected using the EMSA kit (Beyotime Company), according to the manufacturer's instructions (Ahmad et al., 2019).

### Dual-Luciferase Transient Expression System

To examine the expression of the LUC or GUS reporter gene, dual-luciferase transient expression experiments were carried out as described previously (Ahmad et al., 2019). Briefly, the proZmNRT2.1:LUC or proZmNRT2.2:LUC construct was cotransformed with the effector construct pro35S:ZmNLP3.1 as well as proZmUBQ2:GUS into nitrate-free protoplasts. The transformed protoplasts were incubated in nitrate-free solution for 12 h and then treated with 0.5 mM nitrate for 0 or 2 h. After nitrate induction, LUC and GUS activities were measured using a Fluoroskan Finstruments microplate reader (MTX Lab Systems) (Ahmad et al., 2019).

### Identification of Putative *Cis*-Regulatory NREs in *ZmNRT2.1* and *ZmNRT2.2* Promoters

To identify *cis*-acting NREs in the promoter regions of *ZmNRT2.1*and *ZmNRT2.2*, 1 kb sequence upstream of the transcription start site (TSS) of bothgenes was searched using EditSeq (https://www.dnastar.com/). Additionally, MEME(http://meme-suite.org/) was run on -303 to -345 bp and -438 to -480 bp of the *ZmNRT2.1* and *ZmNRT2.2* promoters, respectively. Putative NREs were also identified in the promoters of *NIR* genes of Arabidopsis (*Arabidopsis thaliana*), rice (*Oryza sativa*), spinach (*Spinacia oleracea*), silver birch (*Betula pendula*), common bean (*Phaseolus vulgaris*), and sorghum (*Sorghum bicolor*) using default parameters.

## Results

### ZmCHB101 Regulates Nitrate-Induced Lateral Root Formation and Biomass Accumulation

**Figure 1:**
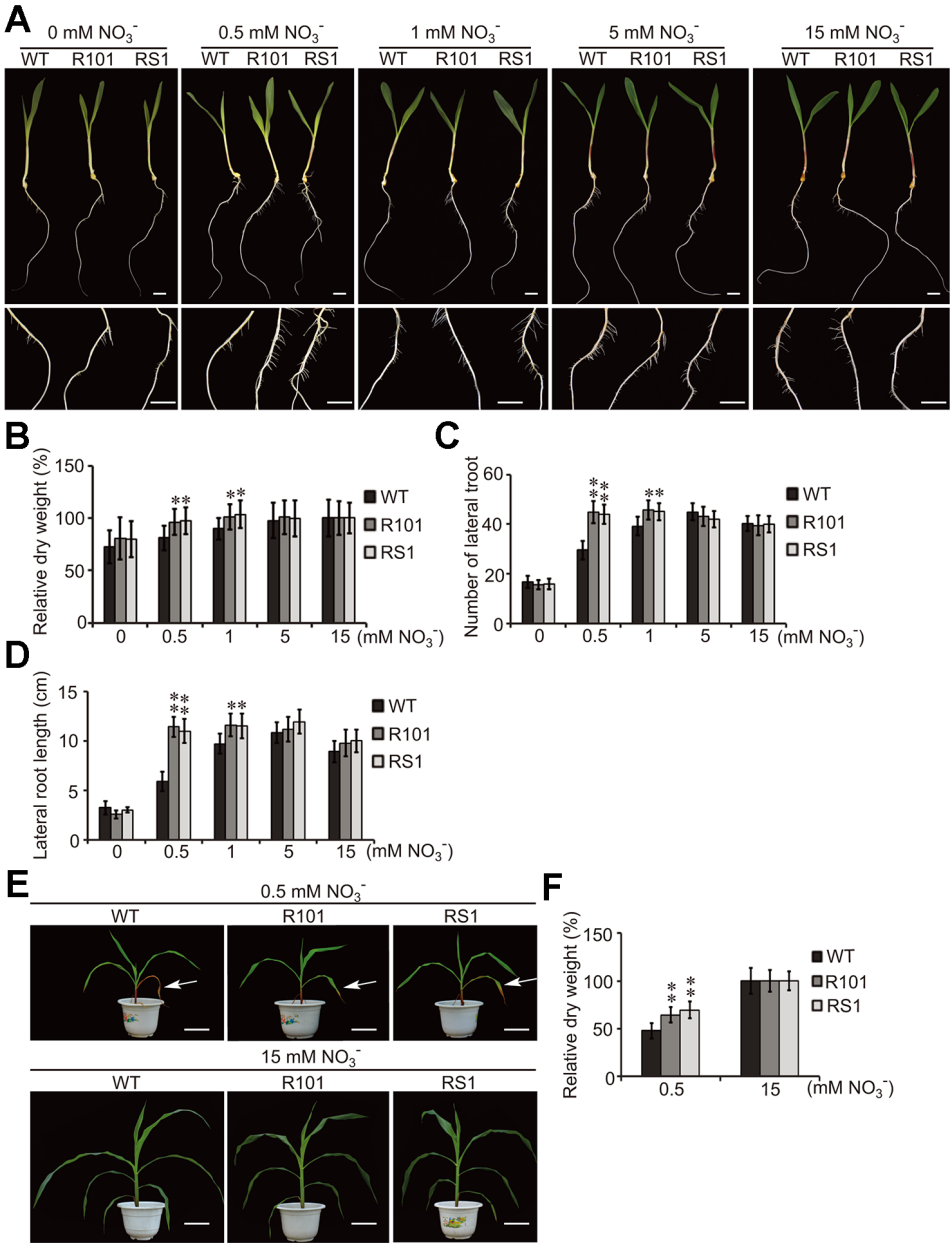
ZmCHB101 plays a negative role in low nitrate response. **(A)** Representative images of seeds at day 5 of nitrate treatment. The germinated seeds were grown in water for 7 days and then transferred to Hoagland's nutrient solution containing 0, 0.5, 1, 5, or 15 mM KNO_3_ for 5 days. **(B–D)** Analysis of the phenotypic traits including the dry weight **(B)**, lateral root number **(C)**, and lateral root length **(D)** of wild-type (WT) and *ZmCHB101-RNAi* plants after 5 days nitrate treatment. Data represent mean ± standard deviation (SD) of three biological replicates. 20 seedlings for each genotype for each biological replicate were used to analysis (*n* = 20). Significant differences are indicated with asterisks (*, *p* < 0.05; **, *p* < 0.01; Student's *t*-test). **(E)** Images of plants after 6 week nitrate treatment. Seedlings were planted in sand without N for 7 days and then watered with nutrient solution containing 0.5 or 15 mM KNO_3_ for 6 weeks. Arrows indicate senescent leaves. **(F)** Dry weight of WT and *ZmCHB101-RNAi* plants measured after 6 weeks nitrate treatment. Data represent mean ± SD of three biological replicates (*n* = 20). Significant differences are indicated with asterisks (**, *p* < 0.01; Student's *t*-test). Data in **(A–D)** demonstrate the short-term effect of different nitrate treatments on plant growth, whereas data in **(E, F)** represent the long-term effects.

Previously, we reported that ZmCHB101 may regulate the expression of genes involved in nitrogen compound metabolic process (Yu et al., 2016). To investigate this possibility, the seeds of WT plants and *ZmCHB101-RNAi* lines were incubated on moist filter paper at 28°C for 3 days to allow germination. The seedlings were then transplanted in pure water and grown for 4 days. To obtain nitrate-free seedlings, after removing the endosperm, the seedlings were transferred to Hoagland's nutrient solution without nitrate for 1 day. Subsequently, 0, 0.5, 1, 5, or 15 mM KNO_3_ was added to the nutrient solution, and lateral root emergence was observed after 5 days. Both *ZmCHB101-RNAi* lines produced a higher number of and longer lateral roots than the WT plants following treatment with 0.1 or 0.5 mM KNO_3_ (**Figures 1A–D**). Notably, these differences between WT plants and *ZmCHB101-RNAi* lines gradually diminished in the presence of 5 or 15 mM KNO_3_ (**Figures 1A–D**).

Next, we planted the seeds of WT and *ZmCHB101-RNAi* lines in sand without N and watered them with nutrient solution containing 0.5 or 15 mM nitrate for 6 weeks. Measurement of the dry weight biomass revealed that the two independent *ZmCHB101-RNAi* lines accumulated a higher biomass than WT plants following the 0.5 mM KNO_3_ treatment (**Figures 1E, F**). Moreover, leaf senescence due to N deprivation was accelerated in WT plants compared with *ZmCHB101-RNAi* lines under the low nitrate conditions (**Figure 1E**). By contrast, there were no discernable phenotypic differences between the WT and *ZmCHB101-RNAi* lines treated with 15 mM KNO_3_ (**Figures 1E, F**). Collectively, these results suggest that ZmCHB101 controls lateral root formation, biomass accumulation, and leaf senescence under low nitrate conditions.

### ZmCHB101 Impacts N Metabolic Processes

**Figure 2:**
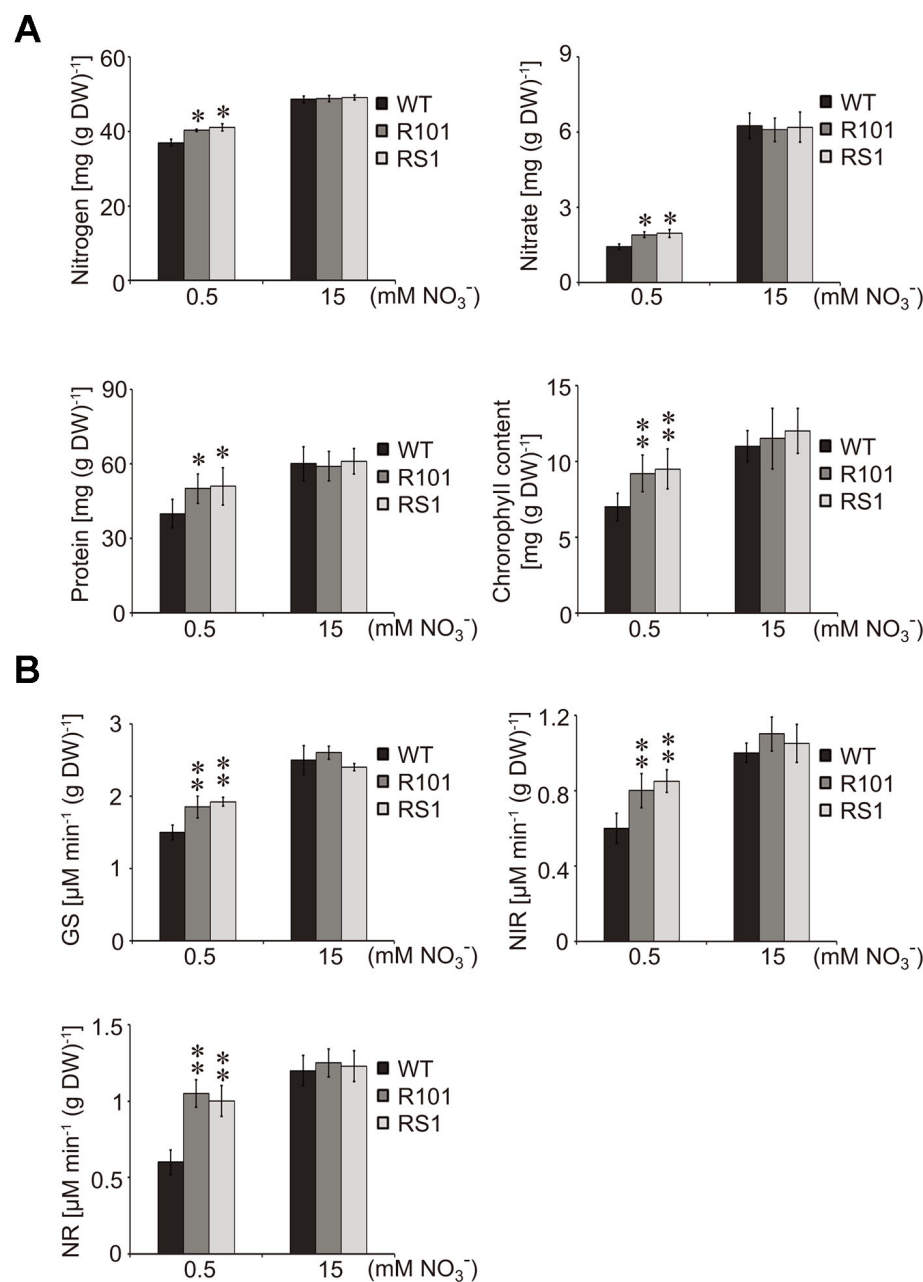
Effects of different concentrations of nitrate on the physiology of maize plants. **(A**, **B)** Analysis of the physiological markers of N status in maize including the contents of N, nitrate, soluble protein, and chlorophyll **(A)** as well as the activities of glutamine synthetase (GS), nitrate reductase (NR), and nitrite reductase (NIR) **(B)**. The WT and *ZmCHB101-RNAi* seedlings were grown in Hoagland's nutrient solution containing 0.5 or 15 mM nitrate for 5 days and used for physiological analysis. Data represent mean ± SD of biological replicates (*n* = 3). Asterisks indicate significant differences (*, *p* < 0.05; **, *p* < 0.01; Student's *t*-test).

Nitrate is an important source of N for amino acid and chlorophyll biosynthesis (Jeuffroy et al., 2002; Hirel et al., 2005). Therefore, we compared various physiological parameters of the WT and *ZmCHB101-RNAi* lines, including the contents of N, nitrate, soluble protein, and chlorophyll, as well as the biochemical activities of the nitrate reductase (NR), nitrite reductase (NiR), and glutamine synthetase (GS) enzymes. Following 0.5 mM nitrate treatment, the total N, nitrate, soluble protein, and chlorophyll contents were significantly higher in the *ZmCHB101-RNAi* lines than in the WT plants (**Figure 2A**). In addition, the activities of NR, NIR, and GS enzymes were also significantly higher in the *ZmCHB101-RNAi* lines than in the WT plants (**Figure 2B**). However, following 15 mM nitrate treatment, these physiological features were similar between the WT and *ZmCHB101-RNAi* lines. These results indicate that ZmCHB101 regulates N metabolic processes under low nitrate conditions.

### ZmCHB101 Regulates the Expression of Nitrate-Responsive Genes

**Figure 3:**
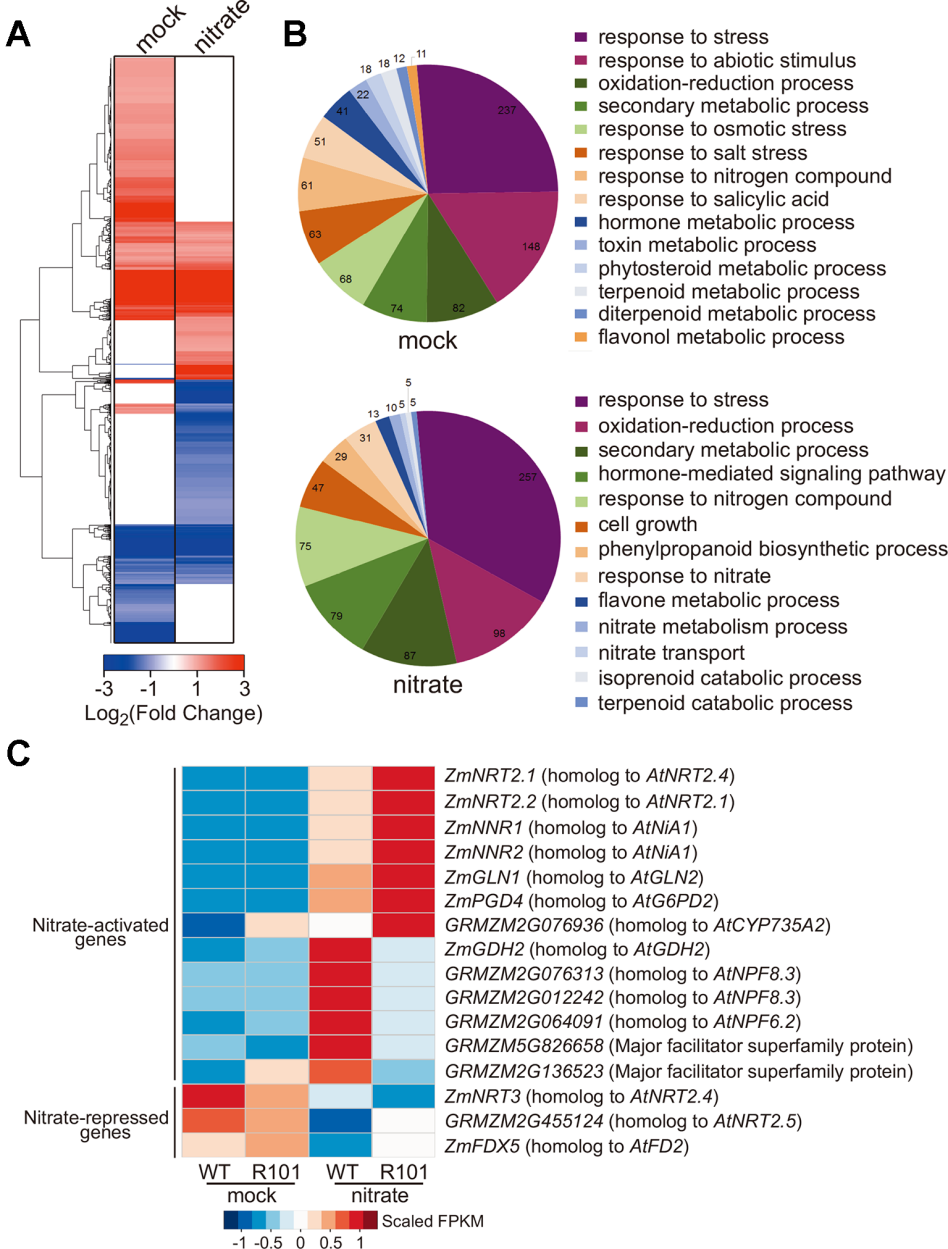
ZmCHB101 regulates transcriptional networks of nitrate-responsive genes in maize roots. **(A**, **B)** Hierarchical clustering analysis **(A)** and Gene Ontology (GO) enrichment analysis **(B)** of genes differentially expressed between 7-day-old nitrate-free WT and *ZmCHB101-RNAi* line R101 seedlings under the mock and nitrate condition. A total of 862 and 786 differentially expressed genes (DEGs) were identified in the WT vs. R101 comparison under the mock and nitrate condition, respectively. The pie charts in **(B)** show significantly enriched GO terms of DEGs. **(C)** Heatmap of DEGs involved in nitrate metabolism. The color scale indicates the FPKM values. Mock, nitrate treatment for 0 h; nitrate, nitrate treatment for 2 h.

To gain insight into the potential role of ZmCHB101 in nitrate-responsive gene expression, we conducted an RNA-Seq analysis of WT and R101 plants treated with 0.5 mM KNO_3_ for 0 (mock) or 2 h (nitrate condition). RNA-Seq data were mapped onto the maize B73 reference genome, and genes that were differentially expressed between WT and R101 plants were identified based on the following criteria: |log_2_FC| > 1 and FDR < 0.05. A total of 862 and 786 differentially expressed genes (DEGs) were identified under the mock and nitrate conditions, respectively (**Figure 3A** and **Supplementary Table S3**). In addition, a gene ontology analysis revealed that a number of biological terms, including “response to nitrogen compound”, “response to stress”, and “response to abiotic stimulus”, were enriched among the DEGs under the mock condition, whereas terms such as “response to nitrate”, “response to nitrogen compound”, “nitrate metabolism process”, and “nitrate transport” were enriched among the DEGs under the nitrate condition (**Figure 3B** and **Supplementary Table S4**).

Next, we categorized the DEGs identified under each condition into two groups: nitrate-activated and nitrate-repressed (**Figure 3C**). Among the nitrate-activated genes, those encoding high-affinity nitrate transporters (categorized as primary nitrate-responsive genes), such as *ZmNRT2.1* and *ZmNRT2.2*, were activated to a higher level in R101 plants than in WT plants (**Figure 3C**). This result indicates that ZmCHB101 negatively impacts the activation of genes encoding high-affinity nitrate transporters. Similar differences in the expression patterns of other nitrate-activated genes were observed between the WT and R101 plants, including *ZmNNR1* and *ZmNNR2* (encoding the nitrate reductase enzymes; (Wang et al., 2004), *ZmGLN1* (*GRMZM2G098290*, encoding the glutamine synthetase enzyme; (Scheible et al., 2004), *ZmPGD4* (encoding glucose-6-phosphate 1-dehydrogenase; (Scheible et al., 2004), and *GRMZM2G076936* (encoding the ortholog of AtCYP735A2; (Takei et al., 2004; Liu et al., 2017) (**Figure 3C**). However, the expression level of *ZmGDH2*, encoding glutamic dehydrogenase 2 (Turano et al., 1997), was significantly increased under the nitrate condition in WT plants, but this induction was dramatically impaired in R101 plants (**Figure 3C**). Similar expression patterns were observed for the *NRT1/PTR* family (NPF) genes *GRMZM2G076313*, *GRMZM2G012242*, and *GRMZM2G064091*, as well as for the major facilitator superfamily proteins related to nitrate/nitrite transport, *GRMZM5G826658* and *GRMZM2G136523* (Sun and Zheng, 2015; Alvarez et al., 2019), all of which had lower expression levels in R101 plants than in WT plants in the presence of nitrate (**Figure 3C**). Among the genes that were down-regulated in the presence of nitrate, repression of *ZmNRT3* was greater in R101 plants than in WT plants, while the reduced expression fold changes of *GRMZM2G455124* (homolog of *AtNRT2.5*) and *ZmFDX5* (homolog of *AtFD2*) were impaired in R101 plants (Scheible et al., 2004; Sabermanesh et al., 2017; Undurraga et al., 2017). The expression patterns of a few selected genes were confirmed by qRT-qPCR (**Supplementary Figure S1**). Overall, these results indicate that ZmCHB101 regulates the expression of nitrate-responsive genes.

### ZmCHB101 Affects Nucleosome Occupancy and Histone Modifications in the Promoters of ZmNRT2.1 and ZmNRT2.2

**Figure 4:**
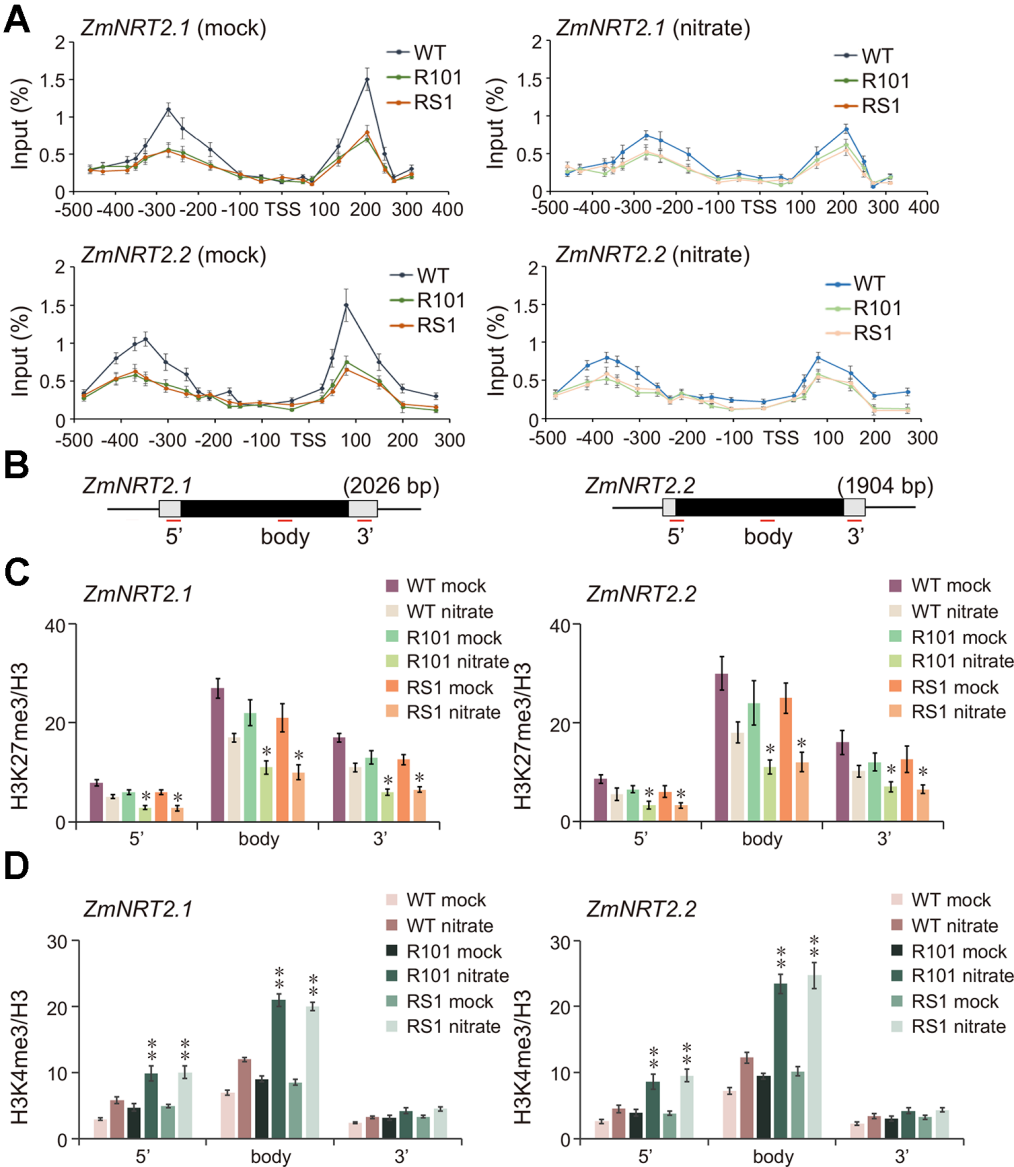
ZmCHB101 affects nucleosome occupancy and histone modification status of *ZmNRT2.1* and *ZmNRT2.2*. 7-day-old nitrate-free seedlings under the mock and nitrate condition were used for chromatin immunoprecipitation and quantitative PCR (ChIP-qPCR) assay. **(A)** ChIP-qPCR using anti-H3 antibody was performed to dissect nucleosome occupancies and densities at -1 and +1 nucleosomes in *ZmNRT2.1* and *ZmNRT2.2* promoters. The X-axis denotes distance from TSS. The Y-axis denotes nucleosome occupancy normalized relative to the input DNA. **(B)** Schematic diagram of *ZmNRT2.1* and *ZmNRT2.2*. The untranslated regions are shown as open boxes and the exons as black boxes. 5', 5' untranslated region; body, gene body region; 3', 3' untranslated region. **(C)** H3K27me3 levels at *ZmNRT2.1* and *ZmNRT2.2*. **(D)** H3K4me3 levels at *ZmNRT2.1* and *ZmNRT2.2.* The Y-axes in **(C**, **D)** denote relative enrichment normalized to the H3. Data represent mean ± SD of the biological replicates (*n* = 3). Mock, nitrate treatment for 0 h; nitrate, nitrate treatment for 2 h. Asterisks indicate significant differences between WT and RS1 or R101 (*, *p* < 0.05; **, *p* < 0.01; Student's *t*-test).

Based on the RNA-Seq results, we speculated that enhanced activation of *ZmNRT2.1* and *ZmNRT2.2* in *ZmCHB101-RNAi* lines under low nitrate conditions may lead to accelerated lateral root formation and higher biomass accumulation. Because ZmCHB101 impacts gene expression by controlling nucleosome density and/or occupancy (Yu et al., 2016), we speculated that nucleosome density and/or occupancy at the *ZmNRT2.1* and *ZmNRT2.2* loci could be impacted in *ZmCHB101-RNAi* lines. To test this possibility, we performed an H3 chromatin immunoprecipitation-coupled with a quantitative polymerase chain reaction (H3 ChIP-qPCR) experiment. Under the mock condition, well-positioned nucleosomes were detected upstream and downstream of the transcription start sites (TSSs; -1 and +1 nucleosome regions) of *ZmNRT2.1* and *ZmNRT2.2* in the WT line, whereas nucleosome densities at these regions were dramatically reduced in the *ZmCHB101-RNAi* lines (**Figure 4A**). Intriguingly, under the nitrate condition, nucleosome densities at the -1 and +1 regions were dramatically decreased in the WT line and remained at a low level in the *ZmCHB101-RNAi* lines (**Figure 4A**). These phenomena were not observed at the promoter regions of *ZmACT1* or *ZmNRT1.1*, a gene encoding a low-affinity nitrate transporter, which was not induced under the nitrate condition (**Supplementary Figure S2A**). Previous studies revealed that well-positioned nucleosomes are also found within the gene body and 3' (near the transcription termination site) regions of expressed genes (Chen et al., 2017; Mueller et al., 2017). In our experiments, the nucleosome densities within the gene body and 3' (near transcription termination site) regions of *ZmNRT2.1* and *ZmNRT2.2* did not differ significantly between the WT and *ZmCHB101-RNAi* lines in either the absence or presence of nitrate (**Supplementary Figures S2B, C**). These results indicate that ZmCHB101 affects the -1 and +1 nucleosome densities of the high-affinity nitrate transporters, *ZmNRT2.1* and *ZmNRT2.2*, but does not alter the nucleosome densities at the gene body and 3' regions of these genes.

A large number of epigenomic analyses have demonstrated that H3K27me3 is associated with strong repression of gene expression, while H3K4me3 is linked to activation of gene expression (Schneider et al., 2004; Turck et al., 2007; Zhang et al., 2007; Vermeulen and Timmers, 2010; Roudier et al., 2011; Sequeira-Mendes et al., 2014; To and Kim, 2014; Wang et al., 2016). Previous studies suggested that *NRT2.1* promoter activity is tightly controlled by H3K27me3 and H3K4me3 in *Arabidopsis* (Bellegarde et al., 2018). Thus, we performed a ChIP-qPCR analysis using anti-H3K4me3 and anti-H3K27me3 antibodies to examine the impact of ZmCHB101 on these two histone modifications. The H3K27me3 levels at the 5', gene body, and 3' regions of *ZmNRT2.1* and *ZmNRT2.2* were slightly lower in the *ZmCHB101-RNAi* lines than in the WT line (**Figures 4B, C**). Nitrate treatment reduced the H3K27me3 levels in the WT plants, and this reduction was even more pronounced in the *ZmCHB101-RNAi* lines (**Figures 4B, C**). By contrast, H3K4me3 levels were moderately higher at the 5', gene body and 3' regions of *ZmNRT2.1* and *ZmNRT2.2* in *ZmCHB101-RNAi* lines than in WT plants (**Figures 4B, D**). Furthermore, the nitrate-induced increase in H3K4me3 levels was greater at the 5' and gene body regions in the *ZmCHB101-RNAi* lines than in the WT line (**Figures 4B, D**).

Next, we examined the binding of ZmCHB101 to the 5', gene body, and 3' regions of *ZmNRT2.1* and *ZmNRT2.2*. To this end, we expressed ZmCHB101-2×FLAG in maize protoplasts and performed a ChIP-qPCR analysis using an anti-FLAG antibody. As shown in **Supplementary Figure S3**, ZmCHB101-2×FLAG, but not FLAG, was strongly associated with the 5' region of *ZmNRT2.1* and *ZmNRT2.2*, but its binding ability became weaker at the gene body and 3' regions. Taken together, these results suggest that ZmCHB101 impacts the nucleosome densities at regions proximal to the TSS and affects the H3K27me3 and H3K4me3 statuses throughout the whole genic regions of *ZmNRT2.1* and *ZmNRT2.2*.

### NREs Are Essential for the Expression of *ZmNRT2.1* and *ZmNRT2.2*

**Figure 5:**
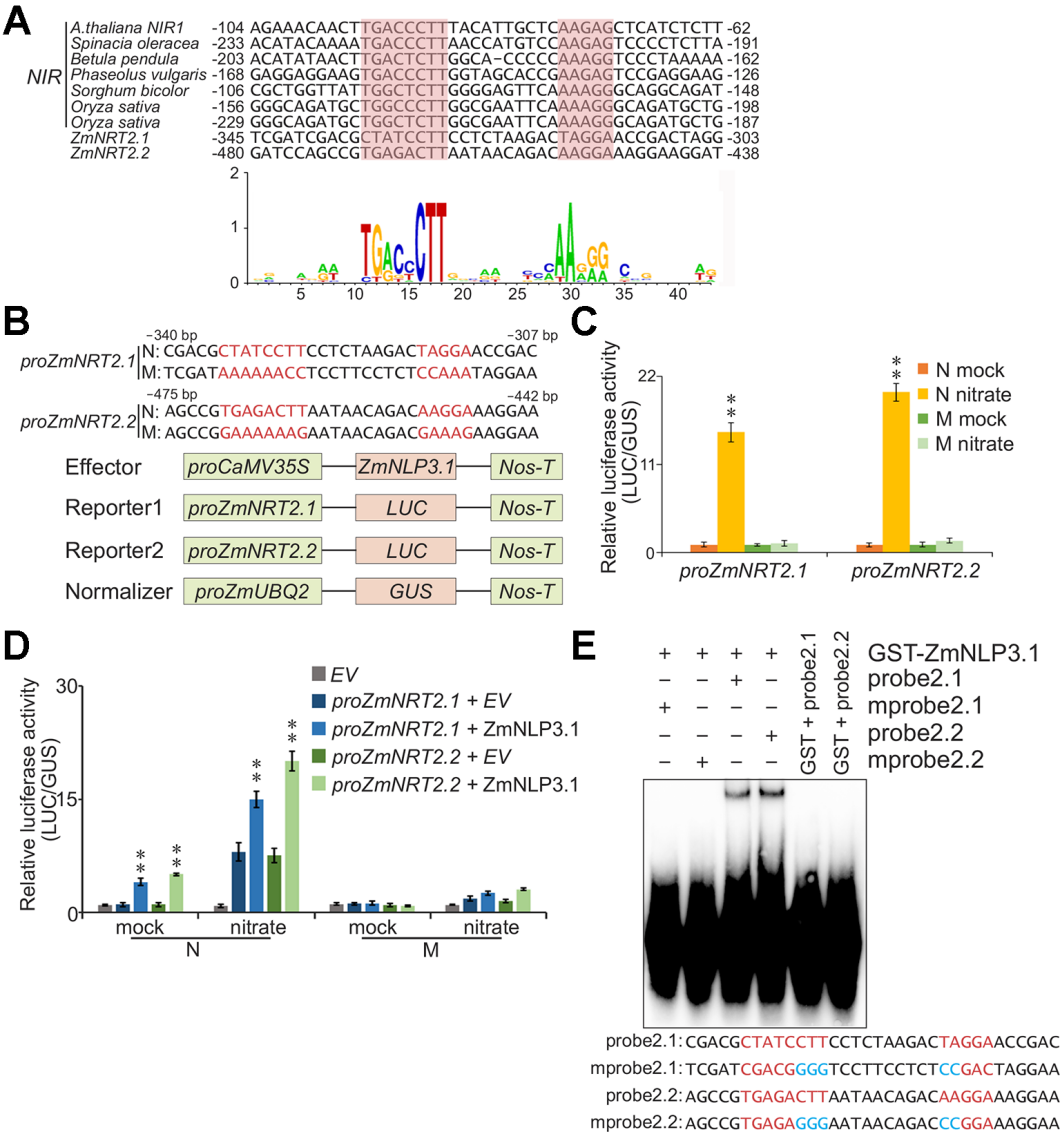
ZmNLP3.1 binds to the promoter regions of *ZmNRT2.1* and *ZmNRT2.2 via* the recognition of NREs and activates gene transcription. **(A)** Nucleotides sequences of nitrate-responsive *cis*-elements (NREs) found in the flanking regions of *ZmNRT2.1* and *ZmNRT2.2*. The NREs of *NIR* genes of *Arabidopsis thaliana*, *Spinacia oleracea*, *Betula pendula*, *Phaseolus vulgaris*, *Sorghum bicolor*, and *Oryza sativa* are indicated. The consensus sequence of NRE is displayed using the sequence logo generation program WebLogo (Crooks et al., 2004). The *p*-values for the prediction of NREs in *ZmNRT2.1* and *ZmNRT2.2* gene promoters were 2.78e-6 and 1.29e-7, respectively. **(B**–**D)** Transcriptional activation of *ZmNRT2.1* and *ZmNRT2.2* by ZmNLP3.1 *via* recognition of the consensus sequence. **(B)** Schematic representation of the intact and mutant NREs in *ZmNRT2.1* and *ZmNRT2.2* promoters. N: normal *ZmNRT2.1* or *ZmNRT2.2* promoter sequence harboring the motif CTATCCTTN_10_TAGAA or TGAGACTTN_10_AAGGA, respectively. M: variants of the *ZmNRT2.1* or *ZmNRT2.2* promoter harboring mutant NREs (AAAAAACCN_10_CCAAA or GAAAAAAGN_10_GAAAG, respectively). **(C)** Nitrate-induced expression of *ZmNRT2.1* and *ZmNRT2.2* genes in protoplasts, depending on the NRE sequences. Nitrate-free protoplasts were transformed with *proZmNRT2.1* or *proZmNRT2.2* and normalizer, incubated for 12 h, and then treated with 0.5 mM nitrate for 0 or 2 h. Mock, nitrate treatment for 0 h; nitrate, nitrate treatment for 2 h. N and M indicate the normal and mutant promoter sequences of *ZmNRT2.1* or *ZmNRT2.2*, respectively, as shown in **(B)**. The ratio of LUC activity to *β-glucuronidase* (GUS) activity was calculated. Data represent mean ± SD (*n* = 3). Asterisks indicate significant differences between mock and nitrate conditions (**, *p* < 0.01; Student's *t*-test). **(D)** Transcriptional activation of *ZmNRT2.1* or *ZmNRT2.2* by ZmNLP3.1 relies on NRE sequences. The *proCaMV35S:ZmNLP3.1* vector was cotransformed with a reporter construct containing either *ZmNRT2.1* or *ZmNRT2.2* promoter and normalizing plasmids in nitrate-free protoplasts. After 12 h incubation, followed by treatment with 0.5 mM nitrate for 0 or 2 h, the LUC and GUS activity was determined. Data represent mean ± SD (*n* = 3). Mock, nitrate treatment for 0 h; nitrate, nitrate treatment for 2 h. Asterisks indicate significant differences between *EV* and ZmNLP3.1 (**, *p* < 0.01; Student's *t*-test). **(E)** Electrophoretic mobility shift assay (EMSA) for analyzing the binding of ZmNLP3.1 to *ZmNRT2.1* and *ZmNRT2.2* promoters. Probe2.1 and probe2.2 denote gene-specific biotin-labeled probes of *ZmNRT2.1* and *ZmNRT2.2* promoters, respectively. In mutant probe 2.1 (mProbe2.1), the sequence CTATCCTTN_10_TAGA in the *ZmNRT2.1* promoter was changed to CGACGGGGN_10_CCGAC. Similarly, in mprobe2.2, the sequence TGAGACTTN_10_AAGGA in the *ZmNRT2.2* promoter was changed to TGAGAGGGN_10_CCGGA.

Since ZmCHB101 regulates nucleosome densities at the promoter regions of *ZmNRT2.1* and *ZmNRT2.2*, we performed a bioinformatic analysis of these promoters using EditSeq (Arnold and Clewley, 1997; Toplak et al., 2012) and detected consensus NREs (5'-GACtCTTN_10_AAG-3'; (Konishi and Yanagisawa, 2010; Konishi and Yanagisawa, 2014) in the promoter regions of both genes (**Figure 5A**). Subsequently, we examined the expression levels of nitrate-responsive genes in nitrate-free maize protoplasts. After 2 h of nitrate induction, key nitrate-responsive genes such as *ZmNRT2.1*, *ZmNRT2.2*, *ZmNNR1*, and *ZmNNR2* were significantly activated relative to the mock condition (**Supplementary Figure S4**). Next, to determine whether the consensus NRE sequence is required for nitrate-responsive gene activation, we co-transfected nitrate-free maize mesophyll protoplasts with *proZmUBQ2:GUS* and the *proZmNRT2.1:LUC* or *proZmNRT2.2:LUC* construct containing normal or mutant NREs in the *ZmNRT2.1* or *ZmNRT2.2* gene promoter. The *proZmUBQ2:GUS* construct was used as a control for evaluating transfection efficiencies. In protoplasts transfected with the normal *proZmNRT2.1:LUC* or *proZmNRT2.2:LUC* construct, the activity of LUC was dramatically higher in the nitrate condition than in the mock condition (**Figures 5B, C**). However, LUC activity was not detected in protoplasts transformed with plasmids containing the mutant form of the *ZmNRT2.1* or *ZmNRT2.2* gene promoter (**Figures 5B, C**).

ZmNLP3.1 plays an essential role in the regulation of nitrate signaling and assimilation processes. It was reported previously that ectopic expression of *ZmNLP3.1* in *nlp7-1* mutant *Arabidopsis* plants restores the N-deficient phenotypes, including shoot biomass, root morphology, and nitrate assimilation under nitrate-replete conditions (Wang et al., 2018). Moreover, nitrate-mediated induction of the *NRT2.1*, *NIA1*, and *NiR1* transcripts is recovered in the *35S::ZmNLP3.1*/*nlp7-1* transgenic lines (Wang et al., 2018). To determine whether ZmNLP3.1 participates in the regulation of *ZmNRT2.1* and *ZmNRT2.2* expression, we co-transfected maize protoplasts with *ZmNLP3.1*, *proZmUBQ2:GUS*, and *proZmNRT2.1:LUC* or *proZmNRT2.2:LUC*. The activity of LUC was greatly induced under the nitrate condition (**Figure 5D**). Intriguingly, LUC activity was higher in protoplasts expressing *ZmNLP3.1* than in those expressing empty vector (**Figure 5D**). These results indicate that ZmNLP3.1 regulates the expression of *ZmNRT2.1* and *ZmNRT2.2* in response to nitrate. Next, we performed electrophoretic mobility shift assays to determine whether ZmNLP3.1 binds directly to the NREs of *ZmNRT2.1* and *ZmNRT2.2*. The full-length GST-tagged ZmNLP3.1 protein (GST-ZmNLP3.1) was capable of binding to probes containing consensus ZmNLP3.1-binding motifs; however, mutations of the NREs in the *ZmNRT2.1* or *ZmNRT2.2* gene promoter abolished the binding of ZmNLP3.1 to these regions (**Figure 5E**). These results indicate that ZmNLP3.1 binds to NREs located in the promoter regions of *ZmNRT2.1* and *ZmNRT2.2*, and activates the expression of these genes in response to nitrate.

### ZmCHB101 Impacts the Binding of ZmNLP3.1 to *ZmNRT2.1* and *ZmNRT2.2* Promoters

**Figure 6:**
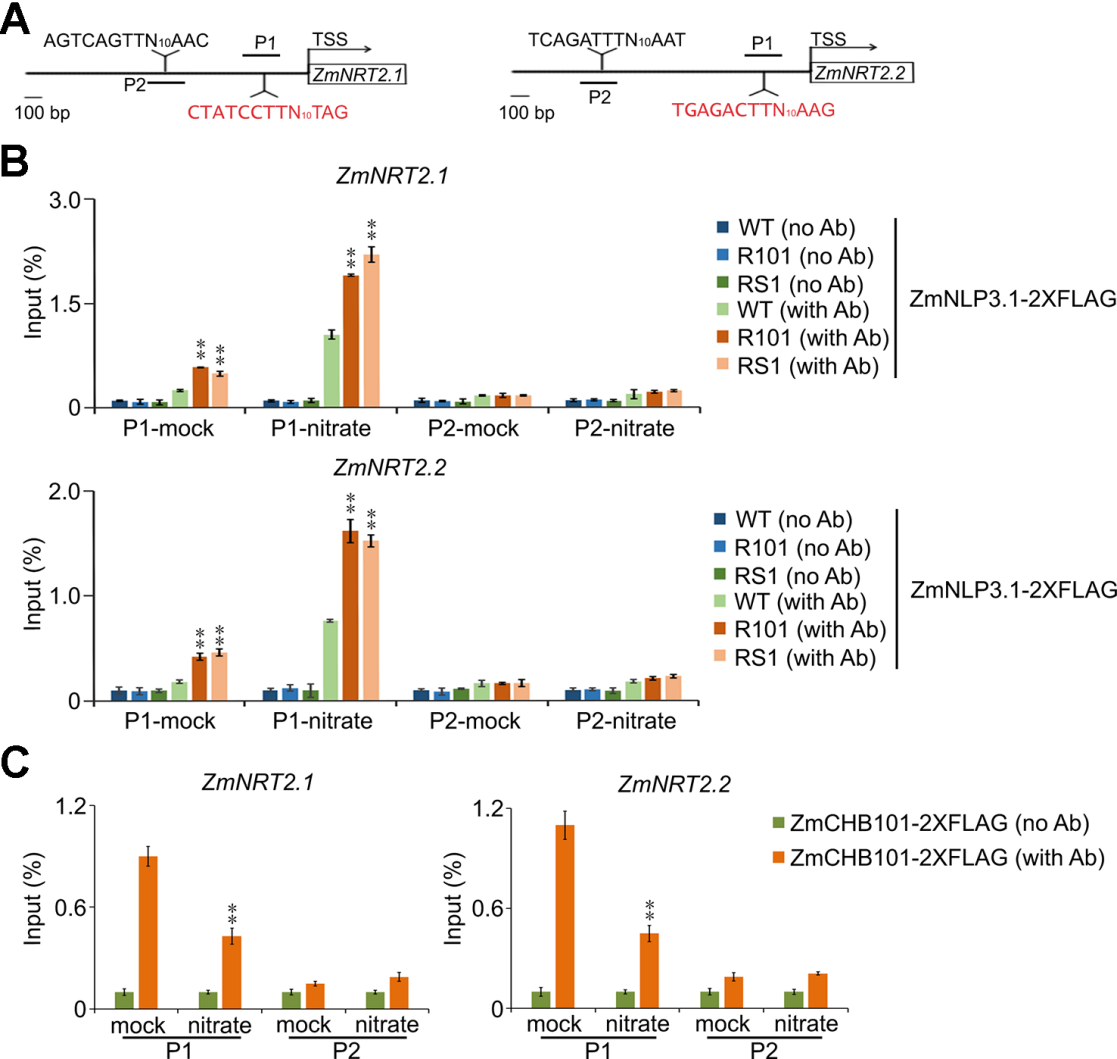
Knockdown of *ZmCHB101* enhances the binding of ZmNLP3.1 to the promoter regions of *ZmNRT2.1* and *ZmNRT2.2*. **(A)** Schematic representation of *ZmNRT2.1* and *ZmNRT2.2* promoters showing the ZmNLP3.1-binding site (P1) and non-ZmNLP3.1-binding site (P2). The NREs located at the -1 nucleosome position are indicted in red. **(B)** The binding of ZmNLP3.1 to NREs in *ZmNRT2.1* and *ZmNRT2.2* promoters was enhanced in *ZmCHB101-RNAi* lines. Nitrate-free WT and *ZmCHB101-RNAi* protoplasts were transformed with *pro35S:ZmNLP3.1-2×FLAG* and then treated with 0.5 mM nitrate for 0 or 2 h. ChIP-qPCR was performed using anti-FLAG antibody. The binding of ZmNLP3.1 to NREs in *ZmNRT2.1* and *ZmNRT2.2* promoters was enhanced in *ZmCHB101-RNAi* protoplasts compared with WT protoplasts. Asterisks indicate significant differences between WT and R101 or RS1 (**, *p* < 0.01; Student's *t*-test). **(C)** Nitrate treatment dissociates ZmCHB101 from the -1 nucleosome position in *ZmNRT2.1* and *ZmNRT2.2* promoters. WT protoplasts were transformed with *pro35S:ZmCHB101-2×FLAG* and then treated with 0.5 mM nitrate for 0 or 2 h. ChIP-qPCR was performed using anti-FLAG antibody. Mock, nitrate treatment for 0 h; nitrate, nitrate treatment for 2 h. Asterisks indicate significant differences between mock and nitrate conditions (**, *p* < 0.01; Student's *t*-test).

**Figure 7:**
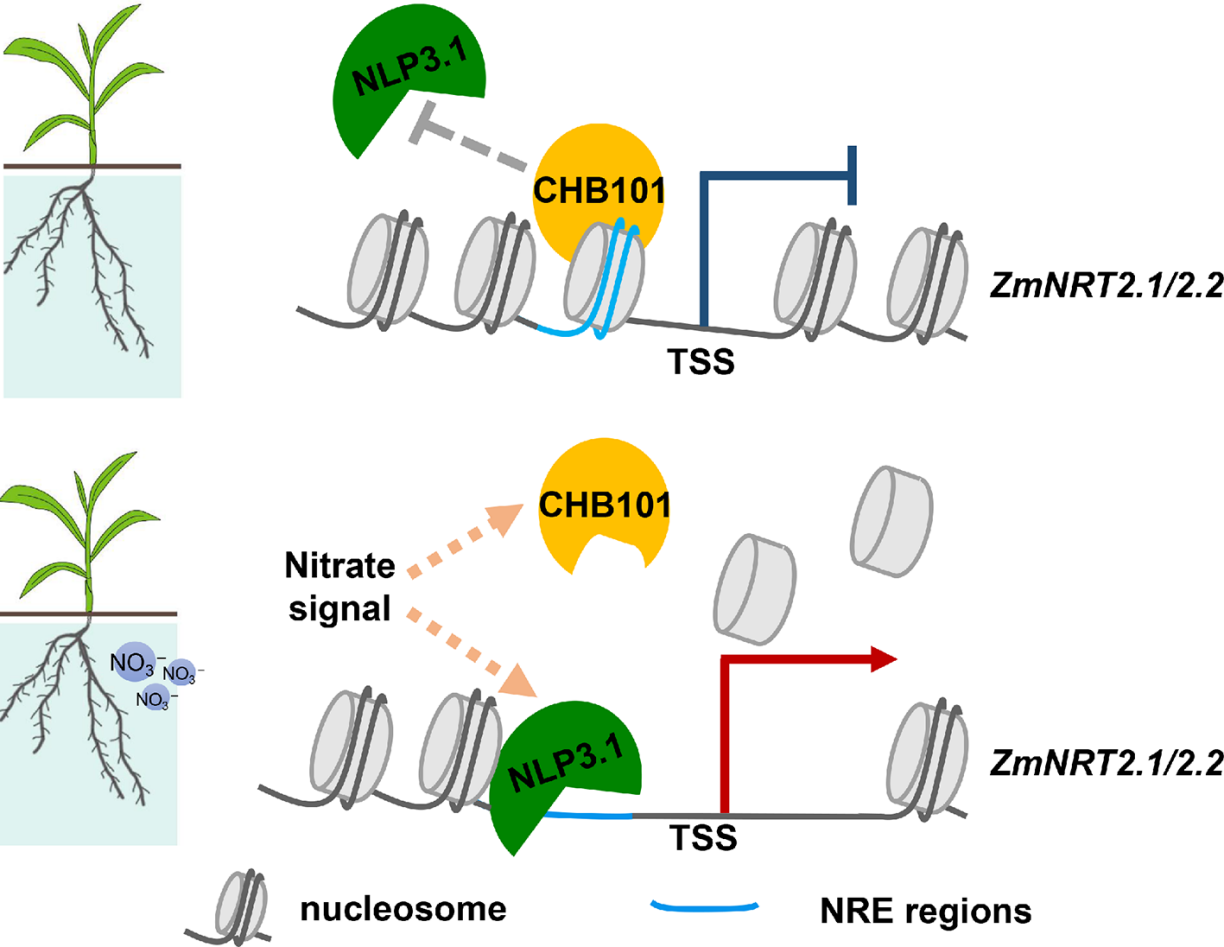
The role of ZmCHB101 in nitrate response in maize. Under nitrate deprivation condition, the ZmCHB101 protein bound to the nitrate-responsive *cis*-elements (NREs) and maintained the nucleosome occupancies at these sites, thereby may impact the binding of ZmNLP3.1 to NREs, and suppresses the expression of *ZmNRT2.1* and *ZmNRT2.2*. In the presence of nitrate, the binding affinity of ZmCHB101 for NREs decreased dramatically, thus decreasing the nucleosome density at NREs and consequently increasing the binding of ZmNLP3.1 to NREs, thus activate the expression of *ZmNRT2.1* and *ZmNRT2.2*.

To determine the molecular interplay between ZmCHB101 and ZmNLP3.1, we transiently expressed ZmNLP3.1-2×FLAG in WT, RS1, and R101 protoplasts, and performed ChIP-qPCR analyses using an anti-FLAG antibody. In the absence of nitrate (mock), ZmNLP3.1 did not bind to NREs (P1) or non-NREs (P2) located in the *ZmNRT2.1* or *ZmNRT2.2* promoter regions of WT protoplasts (**Figures 6A, B**); however, in *ZmCHB101-RNAi* lines, ZmNLP3.1 bound to P1 but not P2 (**Figures 6A, B**). In the presence of 0.5 mM nitrate, ZmNLP3.1 bound to P1 in WT protoplasts, although the level of binding was dramatically higher in the *ZmCHB101-RNAi* lines (**Figures 6A, B**). Subsequently, we performed an additional ChIP-qPCR analysis of ZmCHB101-2×FLAG in WT protoplasts and found that ZmCHB101 could bind to NREs in the absence of nitrate. However, this binding activity was significantly reduced in the presence of 0.5 mM nitrate (**Figure 6C**). Overall, these results indicate that ZmCHB101 impacts the binding of ZmNLP3.1 to NREs *via* an unknown mechanism (**Figure 7**).

## Discussion

Nitrate uptake is a highly regulated process. Maximizing nitrate uptake during seedling development is important because it has a major influence on plant growth and yield. In nature, the concentrations of seed-derived free amino acids in root and shoot tissues are initially high but decrease rapidly until maintaining a constant level 8 days after imbibition. The root nitrate uptake capacity then increases until shoot N content is stabilized (Sabermanesh et al., 2017). One possible method to improve the efficiency of N uptake is to enhance the nitrate uptake capacity of plants because nitrate is the predominant form of N available in the soil in most agricultural areas (Miller et al., 2007). Plant nitrate uptake is mediated by low- and high-affinity transport systems, which are thought to operate at high and low external nitrate concentrations, respectively (Kronzucker et al., 1995; Okamoto et al., 2003; Kotur et al., 2013). In *Arabidopsis*, AtNRT2.1 and AtNRT2.2 mediate high-affinity nitrate uptake; AtNRT2.1 is thought to be responsible for the majority of high-affinity nitrate transport (Li et al., 2007). Following a nitrate starvation period, the high-affinity nitrate transport activities and transcript levels of *AtNRT2.1* and *AtNRT2.2* increase rapidly after replenishing the nitrate supply but are later repressed with prolonged exposure to sufficient nitrate. In this study, *ZmCHB101-RNAi* lines showed enhanced lateral root numbers and biomass accumulation under low nitrate conditions; however, this phenomenon disappeared gradually under high nitrate conditions. In addition, the expression levels of *ZmNRT2.1* and *ZmNRT2.2* were higher in the *ZmCHB101-RNAi* lines than in the WT plants under low nitrate conditions. These results indicate that the high-affinity nitrate transport system is activated more strongly in the *ZmCHB101-RNAi* lines than in the WT line.

Nitrate sensing activates signaling pathways that impinge upon molecular, metabolic, physiological, and developmental responses, both locally and at the whole plant level. However, some gaps still exist in our understanding of how nitrate signaling affects biological processes in plants. Previous studies demonstrated that the SWI/SNF CRC is a central regulatory module in plants that controls biological processes such as cell cycle progression and hormone signaling (Jerzmanowski, 2007; Reyes, 2014; Sarnowska et al., 2016). However, whether the SWI/SNF complex participates in nitrate signaling remains unknown. We showed previously that the ZmCHB101 protein regulates different biological processes in maize, including dehydration stress responses, abscisic acid responses, and shoot and root development (Yu et al., 2016; Yu et al., 2018). In this study, RNA-Seq analyses revealed that ZmCHB101 functions in different biological processes, including “response to nitrogen compound”, “response to stress”, and “response to abiotic stress”. This result, together with the results of previous studies, indicates that ZmCHB101 acts as a general SWI/SNF CRC that participates in different physiological processes. Since we did not have a ZmCHB101-specific antibody, we tried to identify possible targets of ZmCHB101 using RNA-Seq. The expression levels of *ZmNRT2.1* and *ZmNRT2.2*, encoding high-affinity nitrate transporters, were higher in *ZmCHB101-RNAi* lines than in the WT line, identifying them as possible targets of ZmCHB101. Furthermore, ZmCHB101 bound directly to *ZmNRT2.1* and *ZmNRT2.2*, and impacted the chromatin status, indicating that it plays a key role in maintaining nucleosome occupancies at core consensus NREs located in the promoter regions of *ZmNRT2.1* and *ZmNRT2.2* to inhibit their expression. However, upon nitrate induction, ZmCHB101 was likely removed from these NREs, resulting in a dramatic reduction in nucleosome densities at these loci. These results indicate that, while ZmCHB101 maintains nucleosome occupancies at these loci, some unknown nucleosome remodeling factors reduce the nucleosome densities. Reduction of nucleosome densities further facilitates the binding of ZmNLP3.1 to NREs, which activates gene transcription. Since ZmCHB101 and ZmNLP3.1 antibodies are not currently available, we were unable to determine the mechanism by which ZmCHB101 plays a negative role in ZmNLP3.1-mediated gene expression of *ZmNRT2.1* and *ZmNRT2.2*. Further studies are required to elucidate the *in vivo* molecular interplay between ZmCHB101 and ZmNLP3.1 in response to nitrate.

A genome-wide nucleosome occupancy map of maize constructed *via* sequencing of mononucleosomal DNA generated by MNase digestion revealed that nucleosome organization is associated with the plasticity of gene transcriptional status (Chen et al., 2017). The 5' and 3' nucleosome depleted regions become more pronounced as the gene expression level increases (Chen et al., 2017). In addition, the distances between the +1 and -1 nucleosomes and the TSS show a positive correlation with the level of gene expression (Chen et al., 2017). In our current study, the NREs in the promoters of *ZmNRT2.1* and *ZmNRT2.2* were located at -1 nucleosome, indicating that ZmNLP3.1-mediated gene expression is coupled with chromatin remodeling processes. In addition, the *in vivo* binding affinity of ZmNLP3.1 for NREs was dramatically lower in WT plants than in *ZmCHB101-RNAi* lines, both in the absence and presence of nitrate. Moreover, nucleosome densities were dramatically lower in *ZmCHB101-RNAi* lines than in WT plants. Overall, these results indicate that ZmCHB101 is responsible for the maintenance of nucleosomes at NREs in the absence of nitrate. Previously, we proposed that ZmCHB101 is responsible for removing the -1 and +1 nucleosomes from stress-responsive gene promoters (Yu et al., 2018). Because CRCs perform multiple functions, including nucleosome sliding, eviction, and replacement (Clapier and Cairns, 2009), we deduce that ZmCHB101 also plays different roles during transcriptional regulation.

## Data Availability Statement

Data generated in this study are deposited in NCBI Sequence Read Archive (accession number PRJNA541335).

## Author Contributions

Z-YX and BL devised and supervised the project. XM, XY, Z-YX, and BL designed the experiments. XM, XY, YW, DK, NN, WC, and SW performed experiments and analyzed the data. XM, XY, Z-YX, and BL wrote the manuscript.

## Funding

The work was supported by the National Natural Science Foundation of China (#31601311 to Z-YX), the Natural Science Foundation of Jilin Province of China (#20180101233JC to Z-YX), Science and Technology Innovation Development Project of Jilin City (#201831781 to XY), and the Fundamental Research Fund for the Central Universities (#2412018BJ002 to Z-YX) and the National Research Foundation of Korea, Ministry of Science and ICT (#2017R1A4A1015594 and #2017R1C1B2009362 to DK).

## Conflict of Interest

The authors declare that the research was conducted in the absence of any commercial or financial relationships that could be construed as a potential conflict of interest.
