# Supplementary material for: Chromatin Remodeling Protein ZmCHB101 Regulates Nitrate-Responsive Gene Expression in Maize
Source: Front Plant Sci. 2020 Feb 13;11:52. doi: 10.3389/fpls.2020.00052 (PMC7031486; doi:10.3389/fpls.2020.00052)
Supplement: Supplementary file 1 [file Image_1.pdf]

Supplementary Material

Supplementary Data

Supplementary Figure S1. Real-time qRT-PCR analysis of nitrate-responsive genes in WT and *ZmCHB101-RNAi* lines.

Supplementary Figure S2. Nucleosome density at different genic loci.

Supplementary Figure S3. ZmCHB101 directly associates with *ZmNRT2.1* and *ZmNRT2.2*.

Supplementary Figure S4. Real-time qRT-PCR analysis of nitrate-responsive genes in WT protoplasts.

Supplementary Table S1. RNA-seq data information.

Supplementary Table S2. Primers used for different experiments.

Supplementary Table S3. Differentially expressed genes between WT and R101 under the mock and nitrate conditions.

Supplementary Table S4. Go enrichment of differentially expressed genes between WT and R101.

Supplementary Figures

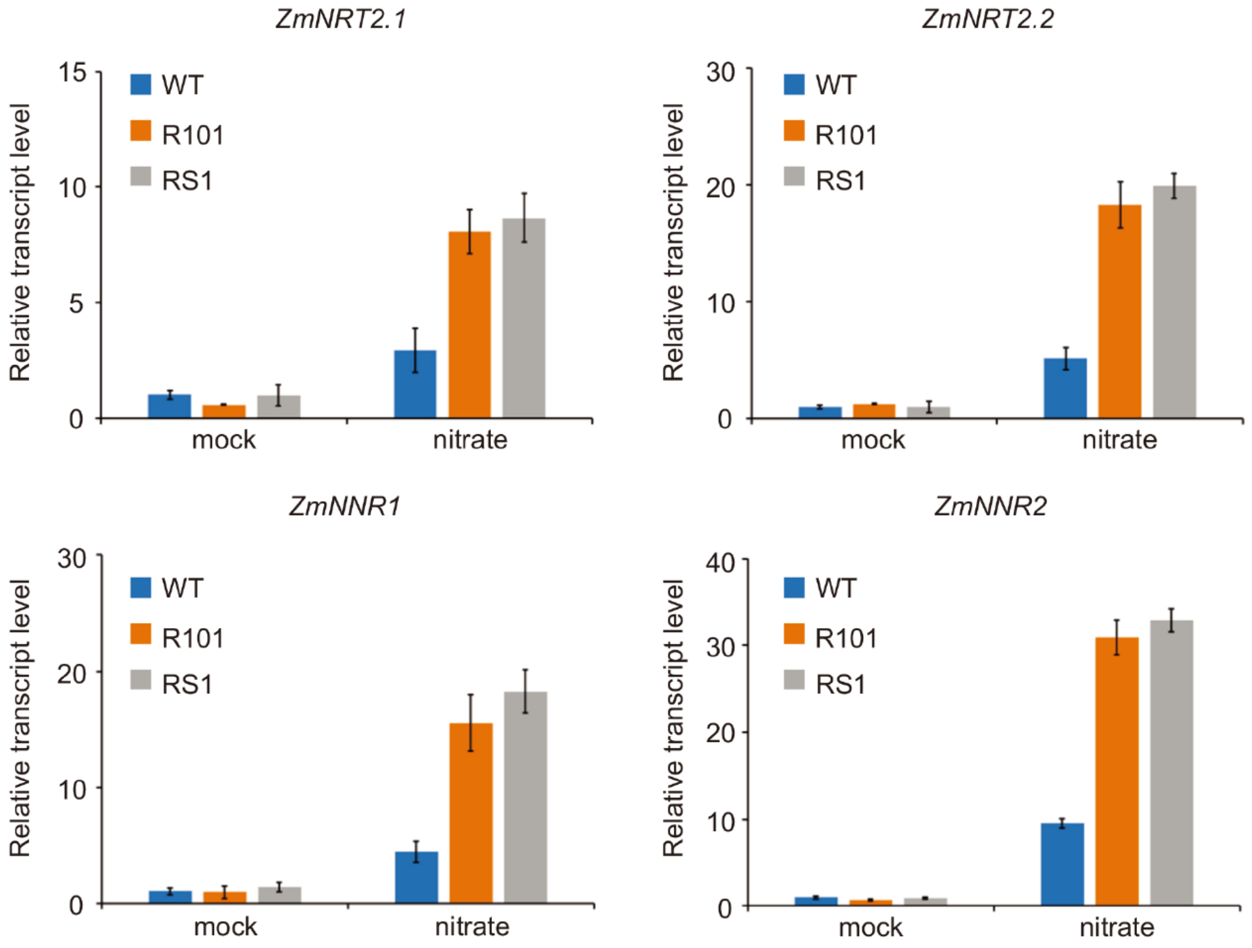

Supplementary Figure S1. Real-time qRT-PCR analysis of nitrate-responsive genes in WT and *ZmCHB101-RNAi* lines. Total RNA was extracted from WT and *ZmCHB101-RNAi* lines roots under 0.5 mM nitrate treatment for 0 h and 2 h. *ZmACT1* was used as an internal control. Error bars indicate SD (n=3). Mock, nitrate treatment for 0 h; nitrate: nitrate treatment for 2 h.

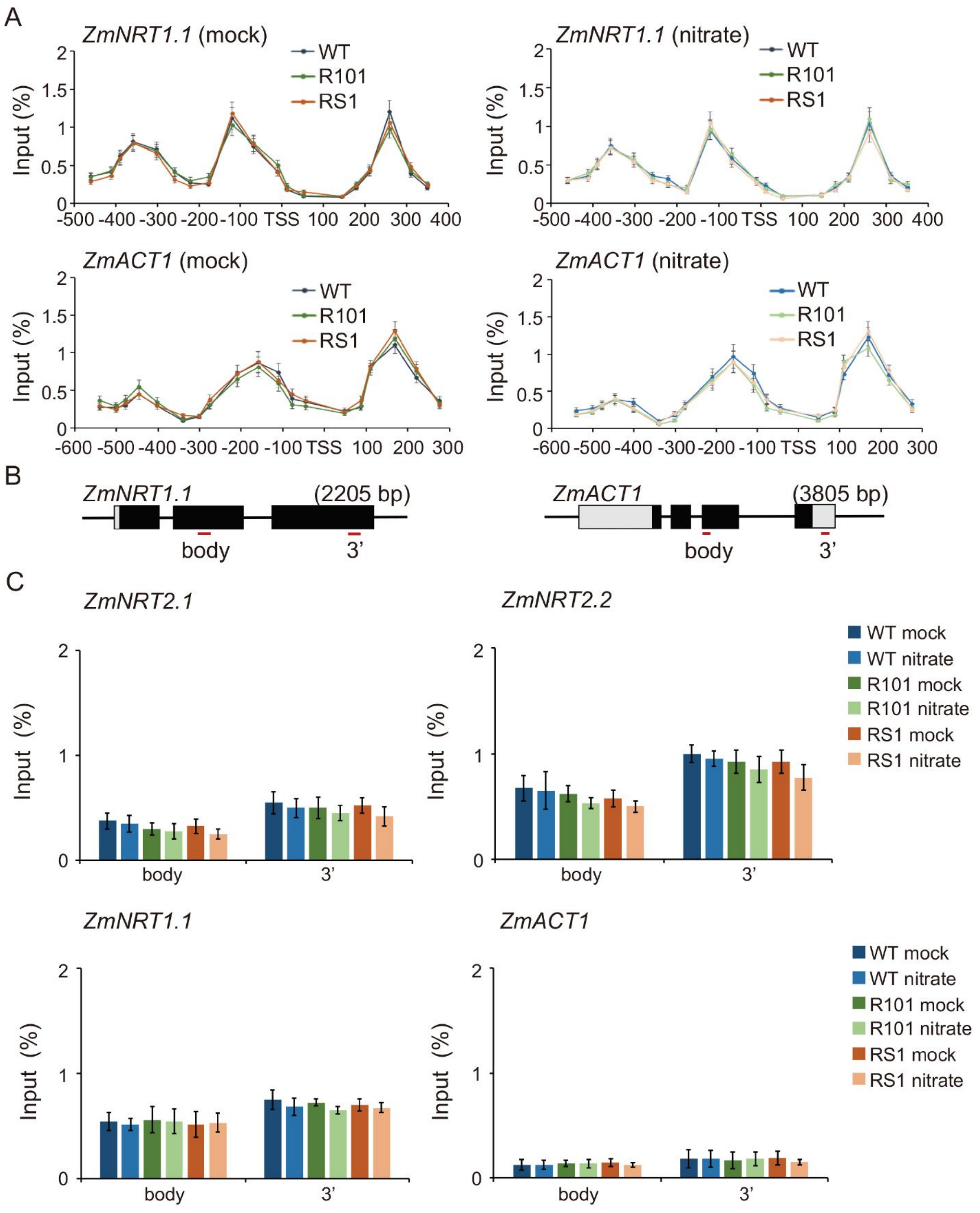

Supplementary Figure S2. Nucleosome density at different genic loci. (A) H3 ChIP-qPCR was performed on -1 and +1 nucleosomes of *ZmNRT1.1* and *ZmACT1*. The X-axis denotes the distance from the transcription start site (TSS), and the Y-axis denotes the relative nucleosome occupancy normalized to the input DNA. Mock, nitrate treatment for 0 h; nitrate, nitrate treatment for 2 h. (B)

Schematic diagram of *ZmNRT1.1* and *ZmACT1*. The untranslated regions are shown as open boxes and the exons as black boxes. body, gene body region; 3’, 3’ untranslated region. (C) Nucleosome densities at body and 3’ regions of *ZmNRT2.1* and *ZmNRT2.2*. Error bars indicate the SD of the biologic replicates (n = 3).

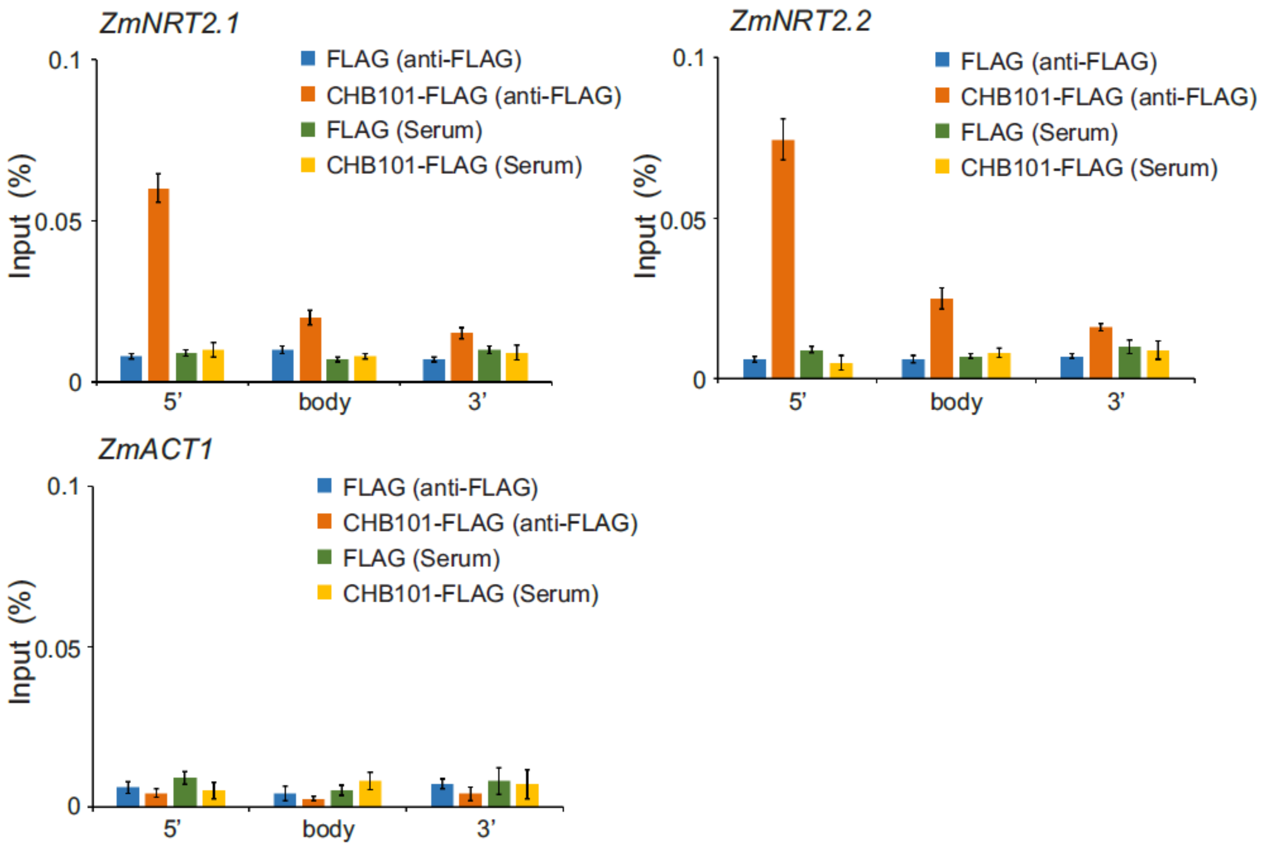

Supplementary Figure S3. ZmCHB101 directly associates with *ZmNRT2.1* and *ZmNRT2.2*. ChIP-qPCR were performed by *ZmCHB101-2×FLAG* or *FLAG* transfected protoplasts using anti-FLAG antibody. X-axis denotes different genetic regions of genes presented in Figure 4B. The Y-axis denotes the relative enrichment normalized to the input DNA.

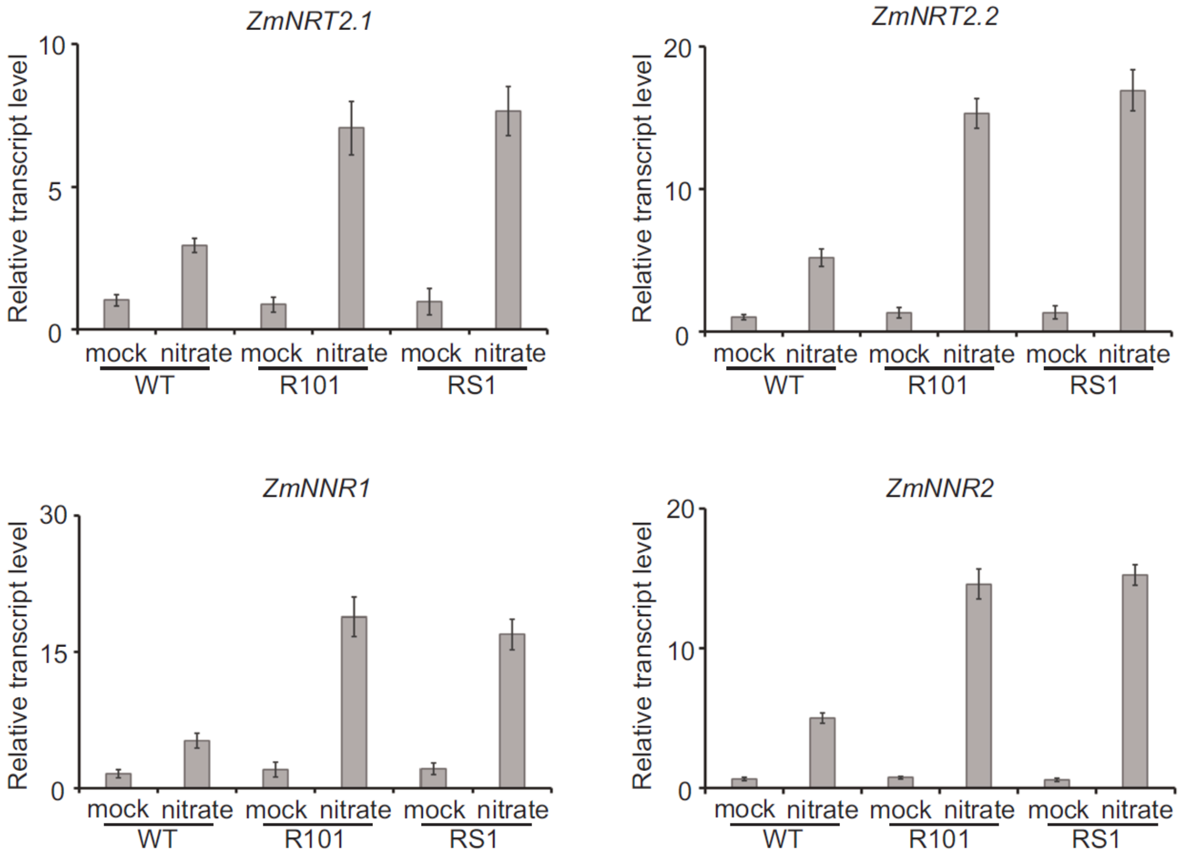

Supplementary Figure S4. Real-time qRT-PCR analysis of nitrate-responsive genes in WT protoplasts. Nitrate free protoplasts were acquired and used for nitrate treatment for 0 h and 2 h. *ZmACT1* was used as an internal control. Error bars indicate SD (n=3). Mock, nitrate treatment for 0 h; nitrate, nitrate treatment for 2 h.
